# Successful laparoscopic transabdominal preperitoneal repair of recurrent inguinal hernia of the bladder following bilayer mesh use: A case report

**DOI:** 10.1016/j.ijscr.2024.110548

**Published:** 2024-10-30

**Authors:** Yusuke Takahashi, Ryoichiro Kobayashi, Hitoshi Seki

**Affiliations:** Department of Digestive Surgery, Nagano Municipal Hospital, Nagano 381-8551, Japan

**Keywords:** Hernia, Inguinal, Urinary bladder, Recurrence, Case report, Laparoscopy

## Abstract

**Introduction:**

The effectiveness of laparoscopic repair for recurrent inguinal hernias has been previously reported; however, recurrence following bilayer mesh use has rarely been reported. We report a case of successful total laparoscopic repair of a recurrent inguinal hernia of the bladder in which a bilayer mesh was used for the initial direct inguinal hernia.

**Presentation of case:**

An 80-year-old man underwent hernia repair using a bilayer mesh for a right direct hernia 12 years ago. Computed tomography revealed a malignant neoplasm of the pancreas and an asymptomatic recurrence of a right direct hernia of the bladder. We performed a laparoscopic distal pancreatectomy and splenectomy for the malignant pancreatic neoplasm. Postoperatively, the patient complained of right inguinal pain and frequent urination. Therefore, 2 months after the pancreatic surgery, we decided to perform laparoscopic repair of the right recurrent inguinal hernia of the bladder. Regardless of severe adhesions attributed to the bilayer mesh in the preperitoneal space, we could safely and definitely complete the laparoscopic transabdominal preperitoneal repair. No postoperative complications were observed, and the patient was discharged on postoperative day 2.

**Discussion:**

In cases of recurrence following hernia repair using a bilayer mesh, both laparoscopic and anterior approaches may be challenging owing to the presence of adhesions.

**Conclusion:**

Definite intraoperative identification of the urinary bladder and mesh placement in the hernia orifice are necessary for an effective laparoscopic approach. Laparoscopic hernia repair may be feasible in cases of recurrence following bilayer mesh use.

## Highlights

• Recurrence following bilayer mesh use has rarely been reported.
• We report successful total laparoscopic repair of recurrent inguinal hernia.
• Laparoscopic hernia repair may be feasible in recurrence following bilayer mesh use.

## 1 Introduction

Inguinal bladder hernia is a relatively rare condition, accounting for less than 4 % of inguinal hernias [1]. The effectiveness of laparoscopic repair for recurrent inguinal bladder hernia has been reported previously [[2], [3], [4]]; particularly, the laparoscopic approach is considered effective when the previous surgery involved the anterior approach [5]. Conversely, when the previous surgery involved the laparoscopic approach, the anterior approach is considered viable in the case of recurrence owing to the absence of adhesion in the preperitoneal space [5]. However, in cases of bilayer mesh use, such as the Prolene hernia system and Ultrapro hernia system (UHS), adhesions occur in the preperitoneal space. Therefore, performing laparoscopic repair for recurrence following bilayer mesh use is challenging and has rarely been reported. We report a case of successful laparoscopic repair using the transabdominal preperitoneal (TAPP) approach for recurrent inguinal bladder hernia following UHS use. This report is in line with the Surgical CAse REport (SCARE) criteria [6].

## 2 Presentation of case

**Fig. 1:**
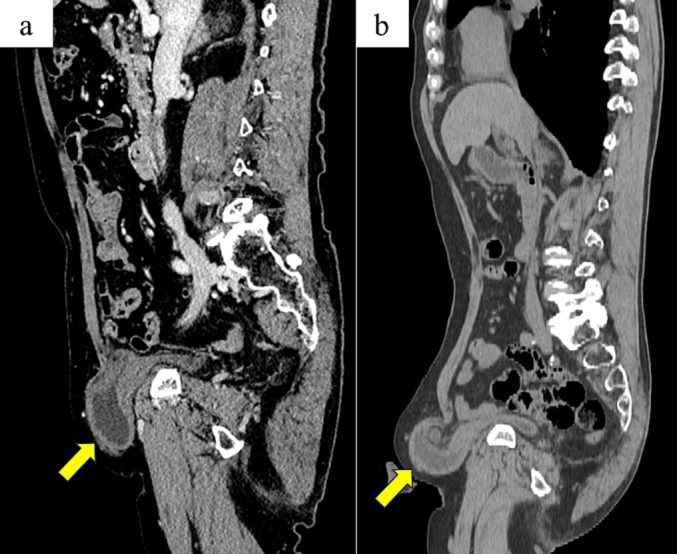
Images on computed tomography (sagittal view). a. Asymptomatic right inguinal bladder hernia (arrow). b. Right inguinal bladder hernia exacerbated (arrow) after pancreatic surgery.

This case report describes an 80-year-old man who underwent right direct inguinal hernia repair (UHS) (Ethicon, Johnson & Johnson, NJ, USA) 12 years previously in our public hospital. A malignant neoplasm of the pancreatic body and an asymptomatic right direct inguinal hernia with urinary bladder hernia content were detected on contrast-enhanced computed tomography (CT) (Fig. 1a). Right bladder hernia recurrence was diagnosed based on the patient's history of hernia repair. We performed a laparoscopic distal pancreatectomy and splenectomy for the malignant neoplasm of the pancreatic body. The pathological diagnosis indicated an invasive intrapapillary mucinous carcinoma. Postoperatively, the patient complained of right inguinal pain and frequent urination. A plain CT image revealed exacerbation of the right bladder hernia upon comparison with the image before pancreatic surgery (Fig. 1b). Therefore, we decided to repair the recurrent right inguinal bladder hernia and perform laparoscopic surgery.

**Fig. 2:**
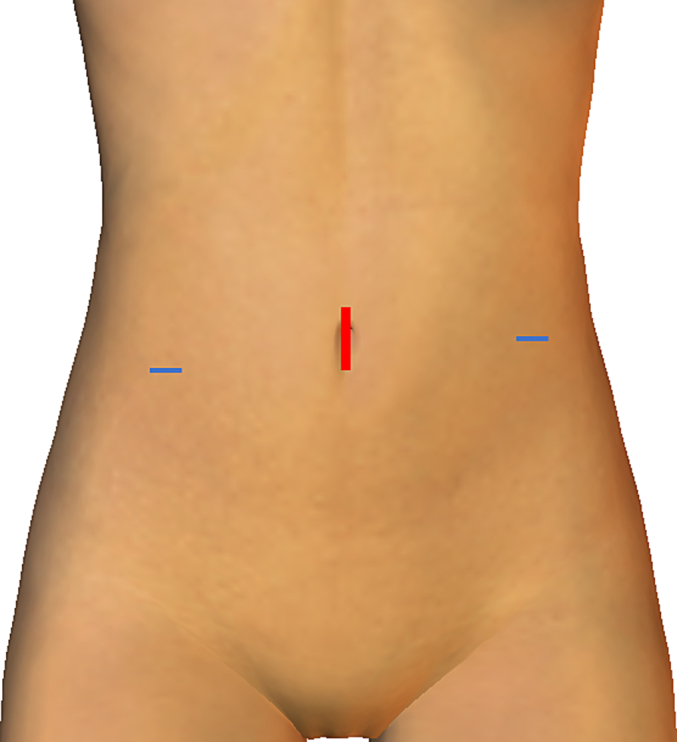
Trocar placement. The red line represents the camera port (12 mm in size) in the umbilicus, and the blue line represents the operator port (5 mm in size). (For interpretation of the references to colour in this figure legend, the reader is referred to the web version of this article.)

**Fig. 3:**
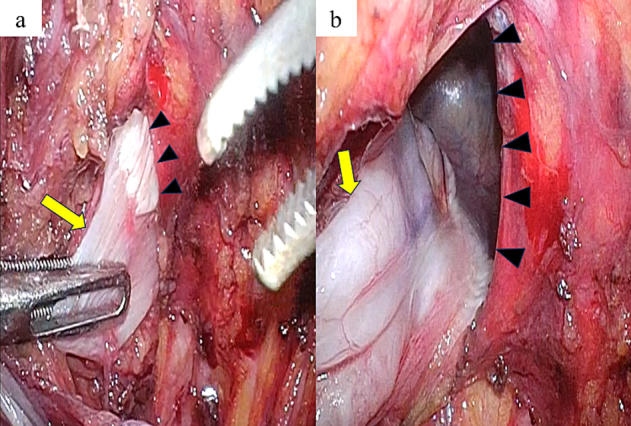
Intraoperative findings. The urinary bladder (arrow) and pseudosac (transverse fascia) (arrowhead) are identified. b. Hernial orifice (arrowhead) is identified. Arrow: urinary bladder.

**Fig. 4:**
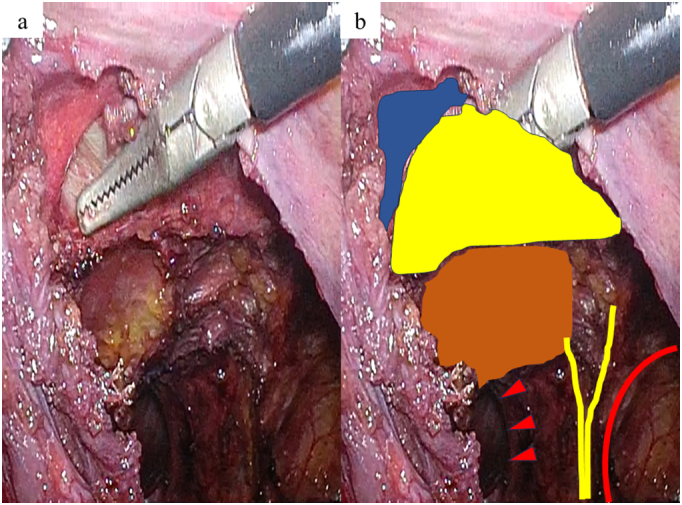
Mesh and anatomical landmark. a. Intraoperative image during dissection between the Ultrapro hernia system (UHS) mesh and medial umbilical fold. b. Colored anatomical landmark. Blue area: medial umbilical fold. Yellow area: UHS mesh. Blown area: abdominal rectus muscle. Yellow lines: inferior epigastric artery and vein. Red line: internal inguinal ring. Arrowhead: hernial orifice. (For interpretation of the references to colour in this figure legend, the reader is referred to the web version of this article.)

Two months after the pancreatic surgery, TAPP repair was performed. Three trocars were placed, as shown in Fig. 2. The border of the urinary bladder and pseudosac (transverse fascia) were easily recognized (Fig. 3a), and the hernial orifice was identified after the urinary bladder was reduced (Fig. 3b). No injury to the urinary bladder was confirmed by injecting 200 mL saline into the urinary bladder. Anatomical landmarks, such as the inferior epigastric vessels, spermatic duct, testicular vessels, medial umbilical fold, Cooper's ligament, and rectus abdominis muscle, were identified; however, severe adhesion between the bilayer mesh and peritoneum was observed (Fig. 4a, b). Right 3D Max® Light mesh (Bard, Murray Hill, NY, USA) was placed in the preperitoneal space and fixed using CAPSURE® (Bard, Murray Hill, NY, USA). The peritoneum was sutured continuously, and the 3D Max® Light mesh was covered. The total surgical time was 2 h 42 min, and blood loss was 2 g. The postoperative course was uneventful, and the patient was discharged on postoperative day 2. No recurrence was observed 1 month later, and the patient showed no complications following the recurrent inguinal hernia repair. Moreover, the patient was greatly satisfied with the outcomes because he was relieved of the inguinal pain and frequent urination.

## 3 Discussion

The present case strongly supports TAPP repair regardless of bladder hernia recurrence and previous bilayer mesh use. Herniation of urinary bladder contents is relatively rare in inguinal hernias, with a reported incidence of less than 4% [1]. It is mostly identified intraoperatively, and lack of preoperative identification can result in bladder injury [7]. Therefore, a detailed preoperative physical examination and definite diagnosis are critical. In the present case, an asymptomatic right bladder hernia was incidentally detected, and its exacerbation after pancreatic surgery was confirmed on CT images. The bladder hernia determined preoperatively by two CT examinations did not result in intraoperative bladder injury. Based on the history of direct hernia repair with UHS, the patient was diagnosed with recurrence. The effectiveness of the laparoscopic approach for initial or recurrent bladder hernias has been reported previously [3,4]. Generally, a laparoscopic approach is considered reasonable for managing recurrence following a previous anterior approach [5]. However, in cases of bilayer mesh use, such as UHS in this case and the Prolene hernia system, which are associated with low recurrence rates (less than 4 %) and comparable postoperative complications compared to other anterior approaches, the laparoscopic approach may be challenging because adhesions can occur in the preperitoneal space and above the transversal fascia [1,[8], [9], [10], [11]]. Hence, an anterior approach may also be considered challenging. Notably, the anterior approach is selected for reoperation in most cases of recurrence after bilayer mesh use [11].

In contrast, the safety and feasibility of “ReTAPP” for recurrence following previous TAPP repair have also been reported [12]. Considering the blind mesh placement in the UHS procedure and the postoperative relief of frequent urination, a blind maneuver related to deconditioning, may have resulted in this recurrence. In the present case, a UHS mesh was placed above the hernial orifice. For definite repositioning of the herniated urinary bladder without injury and precise mesh placement, we considered the laparoscopic approach (TAPP) appropriate. In fact, we could identify the urinary bladder and reduce it without injury; however, adhesion to the UHS mesh was observed. If completing the surgery is considered challenging with the laparoscopic approach, a hybrid (both laparoscopic and anterior) approach may be effective. For example, in case of severe adhesion or intraoperative comorbidities, such as bladder injury and bleeding, conversion to an anterior or hybrid approach could be considered. Total extraperitoneal repair may be impossible because of insufficient access to the preperitoneal space owing to adhesion.

Additionally, important anatomical landmarks, such as the medial umbilical fold, spermatic duct, and testicular vessels, were easily identified owing to the magnified view achieved by laparoscopy, contributing to safer repair. Sufficient coverage of the hernial orifice was also possible because the procedure was performed under the view of the hernial orifice. This precise identification of important anatomical landmarks and the hernial orifice led to a successful TAPP repair. Considering the laparoscopic advantages, this procedure might become first choice.

## 4 Conclusion

TAPP repair may be considered feasible for managing recurrent bladder hernias, even following previous bilayer mesh use. Compared to the anterior approach, TAPP repair ensures safer and more definite mesh repair. In this case, longer follow-up is necessary to precisely evaluate outcomes. Moreover, owing to the limited evidence from published studies, further accumulation of case reports and prospective clinical trials is required to confirm the advantages of TAPP.

## Consent

Written informed consent was obtained from the patient for publication of this case report and accompanying figures.

## Ethical approval

Ethical approval was not obtained for this case report, because this is not clinical research, but case report (Ethics committee, Nagano Municipal Hospital).

## Guarantor

Yusuke Takahashi accepts full responsibility for the work and the conduct of the case report, had access to the data, and controlled the decision to publish.

## Research registration number

This case report was not registered in a publicly accessible database.

## Funding

This research did not receive any specific grant from funding agencies in the public, commercial, or not-for-profit sectors.

## Author contribution

Study concept, design, and writing of this case report were by author: Yusuke Takahashi. RK participated in the treatment of the patients and drafted the manuscript. RK and HS critically revised the manuscript. All authors read and approved the final manuscript.

## Conflict of interest statement

None.
